# Geographical variations in the benefit of applying a prioritization system for cataract surgery in different regions of Spain

**DOI:** 10.1186/1472-6963-8-32

**Published:** 2008-02-04

**Authors:** Rubén Román, Mercè Comas, Javier Mar, Enrique Bernal, Alberto Jiménez-Puente, Santiago Gutiérrez-Moreno, Xavier Castells, the IRYSS Network Modelling Group

**Affiliations:** 1 Evaluation and Clinical Epidemiology Department, Institut Municipal d'Assistència Sanitària (IMAS), Passeig Marítim, 25-29, 08003, Barcelona, Spain; 2 Health Management Department, Hospital Alto Deba (Osakidetza), Avenida Navarra 16, 20500, Mondragón, Spain; 3 Health Services Research Department, Instituto Aragonés de Ciencias de la Salud, Gómez Laguna 25, 50009, Zaragoza, Spain; 4 Evaluation Department, Hospital Costa del Sol, Ctra. Nacional 340 Km 187, 29600, Marbella, Spain; 5 Evaluation and Planification Department, Canarian Health Department, Pérez de Rozas 5, 38004 Santa Cruz de Tenerife, Canary Islands, Spain

## Abstract

**Background:**

In Spain, there are substantial variations in the utilization of health resources among regions. Because the need for surgery differs in patients with appropriate surgical indication, introducing a prioritization system might be beneficial. Our objective was to assess geographical variations in the impact of applying a prioritization system in patients on the waiting list for cataract surgery in different regions of Spain by using a discrete-event simulation model.

**Methods:**

A discrete-event simulation model to evaluate demand and waiting time for cataract surgery was constructed. The model was reproduced and validated in five regions of Spain and was fed administrative data (population census, surgery rates, waiting list information) and data from research studies (incidence of cataract). The benefit of introducing a prioritization system was contrasted with the usual first-in, first-out (FIFO) discipline. The prioritization system included clinical, functional and social criteria. Priority scores ranged between 0 and 100, with greater values indicating higher priority. The measure of results was the waiting time weighted by the priority score of each patient who had passed through the waiting list. Benefit was calculated as the difference in time weighted by priority score between operating according to waiting time or to priority.

**Results:**

The mean waiting time for patients undergoing surgery according to the FIFO discipline varied from 1.97 months (95% CI 1.85; 2.09) in the Basque Country to 10.02 months (95% CI 9.91; 10.12) in the Canary Islands. When the prioritization system was applied, the mean waiting time was reduced to a minimum of 0.73 months weighted by priority score (95% CI 0.68; 0.78) in the Basque Country and a maximum of 5.63 months (95% CI 5.57; 5.69) in the Canary Islands. The waiting time weighted by priority score saved by the prioritization system varied from 1.12 months (95% CI 1.07; 1.16) in Andalusia to 2.73 months (95% CI 2.67; 2.80) in Aragon.

**Conclusion:**

The prioritization system reduced the impact of the variations found among the regions studied, thus improving equity. Prioritization allocates the available resources within each region more efficiently and reduces the waiting time of patients with greater need. Prioritization was more beneficial than allocating surgery by waiting time alone.

## Background

In the last few decades, cataract surgery rates have markedly increased in Western countries. This increase has been due to progressive population aging, improved surgical procedures and broadening of the indication criteria for cataract surgery produced by these improvements [1-4]. Broadening the indication criteria entails that patients with different disability levels can benefit from surgery, modifying the profile of people with unmet needs.

In Spain, the National Health System is decentralized in 17 regions. Each regional health system plans and manages their resources. Important variations in the utilization of health resources have been observed, especially in the elective surgery rate[5]. Studies evaluating the impact of different health policies on the management of need and demand, as well as resource utilization are useful in decision-making[6].

Recently, several health systems have considered the need to prioritize patients on waiting lists, which would entail modification of the current first-in, first-out (FIFO) principle through other models based on need [7-11]. Broadening the indication criteria for cataract surgery entails that the need for surgery differs in patients with appropriate surgical indication. Prioritization of patients by an explicit criterion other than the current FIFO principle would not only avoid unnecessary suffering but it is also expected to reduce the differences between the public demand and the health system utilization in terms of an improved efficiency. In Spain, a recent project has developed prioritization criteria for cataract surgery[12,13]. The objective was to create a prioritization system to ensure shorter waiting times for those patients with greater need, thus increasing the system's efficiency. The resulting prioritization system was obtained using the conjoint analysis technique, and includes clinical (visual impairment and recovery probability), functional (difficulty in doing activities of daily living and ability to work) and social (have someone to look after the patient and be a caregiver) criteria. The most weighted criterion was visual impairment, followed by limitation in doing activities of daily living. Possible priority scores range between 0 and 100, higher scores representing greater need. Thus, in this system, need and priority are equivalent. A pilot study to assess the introduction of the prioritization system in clinical practice was carried out in Catalonia[14] and, Andalusia and Aragon[15].

The effect of introducing a prioritization system would differ in each region because health systems vary widely in terms of clinical practice and utilization rates. Studying these variations is of special interest within the Spanish health system, which provides universal coverage, given that each region manages its own resources.

Simulation techniques can be used to evaluate the impact of introducing a prioritization system in different health management scenarios. Discrete-event simulation (or queuing theory) is an appropriate tool for analyzing waiting lists [16-19], because waiting lists reflect a situation of scarcity and competition for resources, and entries to and exits from the waiting list follow a stochastic law. We defined several hypotheses about what we expected from the simulation model: 1) the prioritization system redistributes the overall waiting time across patients differently than the FIFO system by beneficiating those patients with greater need; 2) differences among regions in the benefit of applying the prioritization system will be due to differences in: surgery Rate, waiting list size and priority score distribution; 3) the model accurately reflects the real system. Several previous experiences have taken advantage of simulation to assess prioritization of demand [17-20] and needs assessment in health services[21,22]. Our objective was to assess geographical variations in the impact of applying a prioritization system in patients on the waiting list for cataract surgery in different regions of Spain, through a discrete-event simulation model.

## Methods

### Discrete-event simulation model

**Figure 1:**
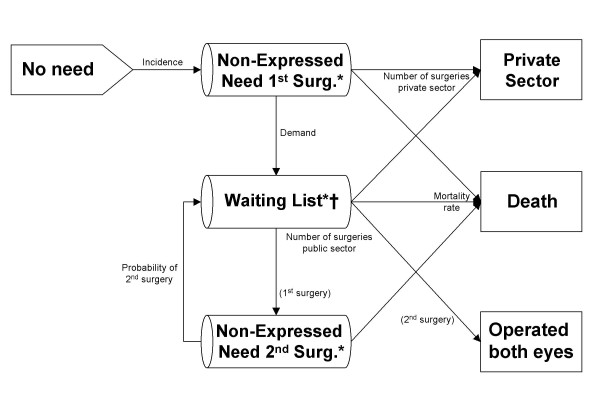
**Conceptual model**. *: Prevalence of need is divided among these 3 states. †: Cases in the waiting list have the priority score as an additional attribute.

A conceptual model to represent the natural process of cataract, from incidence to surgery (Figure 1) was discussed and agreed on by a multidisciplinary expert panel composed of ophthalmologists, epidemiologists, health economists and statisticians. The model referred to individuals from the general population, aged 50 years or older, at risk of need for cataract surgery, and focused on the Spanish health system. The conceptual model was developed by taking into account demand, as well as the particular characteristics, in each of the regions studied: Aragon, Andalusia, Basque Country, the Canary Islands and Catalonia, which represent 45.7% of the Spanish population.

The indication criterion for cataract surgery was defined as any lens opacity causing a visual acuity of 0.5 or less, on a scale from 0 to 1, lower values indicating worse visual acuity[23]. Surgery for this indication was always considered to be appropriate. Need for cataract surgery was defined as meeting the indication criteria for surgery. Incidence was defined as the occurrence of need for surgery.

Need for cataract surgery (Figure 1) was separated into "Non-Expressed Need" and "Waiting List". The state of "Non-Expressed Need" represented the population that, although meeting the indication criteria for surgery, was not included on a waiting list of the Spanish health system. Expressing need was considered equivalent to the following process: requesting surgery and being indicated by a specialist and included on a waiting list of the health system. A distinction was made between first- and second-eye surgery in the "Non-Expressed Need" state (Figure 1), given that senile cataracts are mainly bilateral and interventions are performed in one eye at a time. This distinction was not made in the waiting list, given that the waiting list does not distinguish between patients waiting for first- and those waiting for second-eye surgery. The activity carried out in the private sector was taken into account, given that its activity is high.

### Parameter estimation

The information needed to estimate the model's parameters was compiled for each region studied. Different information sources were used (Table 1), including administrative and research databases. When data from the study's setting were unavailable, data from similar settings was used. The Hospital Discharge Minimum Data Set (HDMDS) of at least three consecutive years was obtained for each of the five regions. This database records all the operations performed in the public sector and allows bilateral operations to be identified. The cataract surgery rates performed in the public sector and the probability of second-eye surgery were obtained from the HDMDS.

**Table 1:** Simulation model parameters, source of information and distribution function.

**Parameter**	**Source**	**Distribution**
**Related to initial state**		
Non Expressed Need 1^st ^Surgery Backlog	North London Eye Study	Fixed value
Non Expressed Need 2^nd ^Surgery Backlog	North London Eye Study	Fixed value
Waiting List Backlog	Waiting list register	Fixed value
Proportion of patients waiting for 2^nd ^eye surgery	Pilot Study (Empirical)	Fixed value
**Static parameters**		
Incident cases per month	North London Eye Study	Poisson*
Number of operations in the private sector per month	Hospital Discharge Minimum Data Set	Poisson*
Proportion of cases of the waiting list who switch to the private sector	Pilot Study (Empirical)	Bernoulli
Top limit for waiting list contents (self-regulation)	Opportunistic	Fixed value
Increase in priority score	Pilot Study (Empirical)	Fixed value
Time between revisions of priority score	Pilot Study (Empirical)	Fixed value
Mortality	Spanish Mortality Register	Empirical lifetime density function
**Dynamic parameters**		
Number of surgeries per month	Hospital Discharge Minimum Data Set	Poisson*
Probability of second eye surgery	Hospital Discharge Minimum Data Set	Bernoulli
Number of bilateral cases entering the waiting list per month	Waiting list register	Poisson*

*Poisson distributions were generated as time between arrivals of the events through an Exponential distribution

Because we used a continuous-time model, the parameters of transitions between states were estimated as distributions of time to an event. Moreover, the possible changes in parameters throughout the 5-year time horizon were taken into account, such as the increase in the number of operations, the probability of second eye surgery[24] or the monthly number of entries to the waiting list (table 1). Since primary data on the prevalence of cataracts in Spain is lacking, a systematic review of prevalence studies of cataracts was carried out[25]. Based on this review the database of the North London Eye Study was used, a population-based study on the prevalence of eye diseases in North London[26]. Prevalence was calculated by age and sex, and its estimates were projected onto the population of each of the five regions studied. In the absence of incidence data, prevalence was used to estimate incidence[27]. The number of inhabitants in each region, as well as the number of deaths by age and sex, in 2001 was obtained from the Spanish National Statistics Institute.

The number of monthly entries to the waiting list in 2003 and the number of patients waiting were obtained from the waiting lists register of each region's health system. The pilot study to assess the introduction of the prioritization system in clinical practice with data from Catalonia[14], Andalusia and Aragon[15] was used to calculate the distributions of priority score at entry to the waiting list, as well as the proportions of patients with bilateral cataract or aphakia (those who had already undergone surgery in one eye). Different empirical distributions were used for bilateral and aphakic patients because statistically different scores were found for first- and second-eye surgery. In the absence of priority data for the Canary Islands and the Basque Country, in these two regions we used a pooled priority distribution of the three regions for which priority data was available. As the prioritization system included clinical and functional criteria that may worsen over time, an increase in priority score with time was evaluated and introduced. Table 1 summarizes the parameters introduced in the model and their sources of information and distribution functions.

Geographical variation was measured through rates (number of occurrences per 100,000 inhabitants), high/low ratio for rates, and coefficient of variation, defined as the ratio of the standard deviation relative to the mean.

### Simulation

The conceptual model (Figure 1) was implemented as a discrete-event simulation model in the SIMUL8 v.10 package (SIMUL8 Corporation)[28] and was run with the corresponding data from each region. The time units were months and the simulation horizon was 60 months (5 years). Each patient was assigned a set of attributes, including age, sex, priority for first- and second-eye surgery (when applicable), "type" of patient (bilateral or aphakic) and lifetime. The priority scores were generated when a patient entered the waiting list and took into account whether the patient had bilateral cataracts or aphakia.

As the impact of the time waited depends on the level of need, the measure of results used as the main outcome was waiting time weighted by priority score, which can be interpreted as the time that a patient waits, due to the waiting list, weighted by his/her need for surgery. This measure allowed waiting times to be compared by taking into account how these times were assigned according to each patient's priority level. Thus, the difference between two simulations could be interpreted as the time, weighted by need, saved or lost with the prioritization system versus the FIFO discipline. The waiting time weighted by priority score included all patients who entered the waiting list: those undergoing surgery, those who were still waiting at the end of the simulation period, those who switched to the private sector, and those who died while on the waiting list. Trials were performed including 20 independent replications, each beginning with the same initial conditions. This sample size was calculated to obtain sufficient precision for comparison between waiting list disciplines[16]. The analyses were based not only on the waiting time weighted by priority score, but also on the raw waiting time of patients. Different thresholds of priority score according to eventual fixed guarantee times were calculated. These thresholds indicated the minimum priority score needed to be operated under a given guarantee time

## Results

The expert panel evaluated the model's results and considered them to be valid and credible. Different patterns of aging were found among regions: Aragon, Catalonia and the Basque Country showed the greatest ageing, with more than 34% of their populations being over 50 years of age. In Andalusia and the Canary Islands, less than 30% of the population was over 50 years old. The estimated percentage of the population with need for cataract surgery was between one-fifth and one-fourth of the population over 50 years of age in all the regions studied (Table 2).

**Table 2:** Descriptive information on senile cataracts in the autonomous regions studied.

	**Regions**				
	
	**Andalusia**	**Aragon**	**Basque Country**	**Canary Islands**	**Catalonia**
**Population**	7,357,558	1,204,215	2,082,587	1,694,477	6,343,110
Population Over 50 years	2,142,202	457,631	744,419	449,819	2,164,467
% Population Over 50 years	29%	38%	36%	27%	34%
**Prevalence**					
% Prevalence in people over 50 years	22.4%	25.8%	22.8%	20.4%	23.5%
Surgery rate *					
Yearly rate	405	529	607	440	685
Surgery rate in people over 50 years	1,391	1,392	1,724	1,650	2,156
**Waiting List**	9,205	2,826	2,313	5,771	19,586
% of prevalent population	1.9%	2.4%	1.4%	6.3%	3.8%
Waiting List entry rate (2003) *	612	755	656	602	733
**Mean priority (at entry to the waiting list)**					
First surgery (SD)	47.1 (19.9)	28.3 (22.4)	39.3 (22.7) ^†^	39.3 (22.7) ^†^	36.5 (22.8)
Second surgery (SD)	36.8 (22.3)	13.7 (11.7)	28.8 (22.6)^†^	28.8 (22.6)^†^	26.1 (22.2)

* N° of ocurrences/100,000 inhabitants

^† ^A pooled Distribution was used in the absence of empirical data

SD: Standard Deviation.

A coefficient of variation (COV) of 0.24 was found in surgery rates among the regions studied. In particular, the surgery rates found in Catalonia were greater than those in the Canary Islands and Andalusia (high/low ratio 1.76 and 1.69 respectively). The rates of entries to the waiting list were more homogeneous among regions than the surgery rates (COV: 0.1). The percentage of the prevalent population included on a waiting list was less than 6.5% in all regions. This percentage varied among the regions studied (COV: 0.62), Table 2. The results of the pilot study [14,15] showed significant differences in the mean priority score at entry to the waiting list among the three regions for which data were available (data not shown). Priority scores showed a dispersion that covered the entire range of possible values. The 25th and 75th percentiles of the assigned priority scores were 34 and 62 points respectively for first-eye surgery and 20 and 53 points for second-eye surgery in Andalusia, 7 and 46 for first-eye surgery and 6 and 21 for second-eye surgery in Aragon, and 20 and 52 for first-eye surgery and 6 and 41 for second-eye in Catalonia.

Simulation of the current waiting list scenario (FIFO) showed that the raw mean waiting time of patients who underwent surgery in the public sector varied from 1.97 months (95% CI 1.85; 2.09) in the Basque Country to 10.02 months (95% CI 9.91; 10.12) in the Canary Islands, Table 3. When the prioritization system was applied, the mean waiting time was reduced to 0.73 months weighted by priority score (95% CI 0.68; 0.78) in the Basque Country (lowest value) and 5.63 months (95% CI 5.57; 5.69) in the Canary Islands (highest value), Table 3. However, patients still waiting at the end of the simulation period had longer waiting times with the prioritization system than with the FIFO discipline. Differences of 11.3 raw months (95% CI 9.4; 13.3) in Andalusia, 4.7 months (95% CI 4.3; 5.1) in Aragon, 5.8 months (95% CI 5.3; 6.4) in the Basque Country, 12.4 months (95% CI 11.0; 13.7) in the Canary Islands and 6.9 months (95% CI 6.2; 7.6) in Catalonia were found. Patients who died while on the waiting list also had longer mean waiting times with the prioritization system than with the FIFO discipline, with waiting times increased by 8.3 months (95% CI 7.3; 9.4) in Andalusia, 4.4 months (95% CI 3.9; 4.8) in Aragon, 5.3 months (95% CI 4.7; 5.9) in the Basque Country, 8.8 months (95% CI 8.0; 9.6) in the Canary Islands and 5.8 months (95% CI 5.2; 6.5) in Catalonia.

**Table 3:** Raw waiting times (FIFO) and times weighted by priority score (FIFO, prioritization system)

			**Waiting times weighted by priority score**					
			
	**Raw waiting times (FIFO)**		**FIFO System**		**Prioritization system**		**Benefit of the priorization system**	
				
	**Mean**	**SD**	**Mean**	**SD**	**Mean**	**SD**	**Mean**	**SD**
Andalusia	2.91	[0.09]	2.81	[0.09]	1.69	[0.11]	1.12	[0.08]
Aragon	5.19	[0.21]	4.89	[0.18]	2.16	[0.12]	2.73	[0.12]
Basque Country	1.97	[0.27]	1.93	[0.26]	0.73	[0.11]	1.20	[0.16]
Canary Islands	10.02	[0.24]	7.23	[0.21]	5.63	[0.13]	1.60	[0.16]
Catalonia	4.48	[0.57]	4.26	[0.52]	1.99	[0.36]	2.27	[0.20]

FIFO: First-in, first-out. SD: Standard Deviation.

The overall mean waiting time weighted by priority score, that is, considering each patient who entered the waiting list (operated patients, patients still waiting at the end of the simulation period, patients who switched to the private sector and patients who died while on the waiting list), was reduced in all the regions when the prioritization system was applied. The waiting time weighted by priority score saved by the prioritization system was 1.12 months (95% CI 1.07; 1.16) in Andalusia, 2.73 months (95% CI 2.67; 2.80) in Aragon, 1.20 months (95% CI 1.11; 1.28) in the Basque Country, 1.60 months (95% CI 1.51; 1.69) in the Canary Islands and 2.27 months (95% CI 2.17; 2.38) in Catalonia, Table 3.

**Figure 2:**
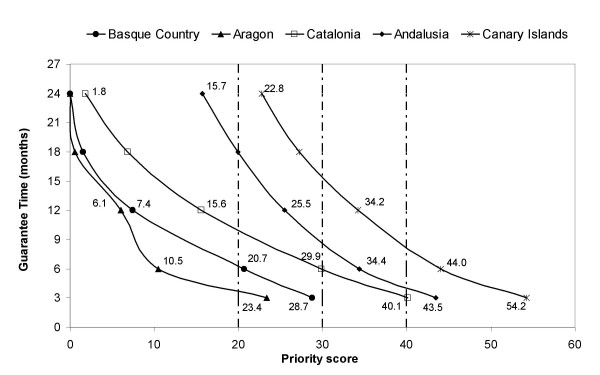
Minimum priority scores for guarantee times.

Figure 2 shows the relationship between the priority score and the waiting time under the prioritization system, i.e., the minimum priority score required for a patient to undergo surgery under an eventual guarantee time, fixed at 3, 6, 12, 18 and 24 months. In patients with a priority score at entry to the waiting list of 40 or more points, the maximum guarantee time was 4 months in Andalusia, 1 month in Aragon, 1 month in the Basque Country, 8.5 months in the Canary Islands and 3 months in Catalonia. In addition, as the priority score at entry in the waiting list diminished, the maximum guarantee time increased. Patients with less than 20 points waited 18 months or more in Andalusia, more than 4 months in Aragon, more than 6 months in the Basque Country, more than 24 months in the Canary Islands, and more than 10 months in Catalonia. A decreasing trend was observed in differences in waiting time among regions as priority scores increased.

When the prioritization system was applied the waiting time range among regions was reduced in 10.64 months for patients within the 20–29 priority score interval with respect to patients in the 60–69 priority score interval, Table 4. Under the current waiting list scenario (FIFO) there was no reduction in the waiting time range among regions. This range held constant around 8 months among all the priority score groups, Table 4.

**Table 4:** Maximum and minimum waiting time weighted by priority score for given priority scores (FIFO and prioritization system)

	**Waiting times weighted by priority score**					
	
**Priority Scores**	**FIFO System**			**Prioritization System**		
		
	Maximum*	**Minimum**^†^	**Difference**	Maximum*	**Minimum**^†^	**Difference**
20–29	10.01	1.96	8.05	11.32	0.54	10.78
30–39	10.01	1.97	8.04	2.75	0.17	2.58
40–49	10.01	1.97	8.04	1.01	0.08	0.93
50–59	10.02	1.97	8.05	0.34	0.04	0.3
60–69	10.02	1.97	8.05	0.17	0.03	0.14

* Maximum waiting times belong to the Canary Islands (see table 3)

^† ^Minimum waiting times belong to the Basque Country (see table 3)

## Discussion

The model described allows several factors commonly used separately by decision-makers to be integrated into a complex but understandable system. Our findings show firstly that introducing a prioritization system improved the impact of cataract procedures by minimizing waiting time in patients according to their level of need and secondly that the benefit of applying the prioritization system varied substantially, depending on the specific characteristics of each region's local health system.

To measure the impact of waiting in accordance with patient's need, the waiting time weighted by priority score was used. Although this measure was based on individual data, it can be interpreted as a global measure of benefit since it took into account the priority scores of all patients who had been assigned a priority score. This measure is based on patients' need and not on health benefit. Unpublished analyses on the prioritization system showed that correlation between the priority score and the utility questionnaires EQ-5D and HUI-3 is low (0.1 and 0.15, respectively). As there is no evidence of relationship between need (priority) and benefit (utility), results were based on need only. The prioritization system reduced the waiting time up to half the time under the actual FIFO discipline (table 3). The waiting time was not measured at a fixed time point; instead it was measured as the average waiting time throughout the time horizon for all patients. Application of the priority system redistributed the total time waited across patients. The model shows how patients with greater need waited less than those with low levels of need. Previous experiences have concluded that assigning surgery according to priority criteria is more beneficial than assignation by waiting time [17-19]. Although the prioritization system was beneficial as a whole, patients with low priority scores had very long waiting times. However, application of the prioritization system should guarantee a maximum waiting time to these patients. Dunn et al.[29] showed that 80% of patients rated waits of 3 months or less as acceptable, while 25% regarded waits of 6 months or longer as too long. Moreover, patients with greater disability were those less tolerant with waiting times.

Several studies have observed geographical variations in clinical practice worldwide[30]. Most of the results found in other countries can be extrapolated to Spain, which offers universal coverage. In agreement with previous studies[31,32], the variation in clinical practice found among regions is notable. Nevertheless, the reasons for this variation are difficult to identify. A small percentage could be explained by demographic and morbidity characteristics of the populations but the main reasons are management features and the availability of resources. The results obtained suggest that prioritization systems reduce geographical variations in waiting time in patients with higher levels of need, that is, in those with high priority scores. Differences among regions in the overall waiting times were reduced when applying the prioritization system. The overall rank between the regions with the maximum and the minimum mean waiting time is reduced from 8.1 months under the FIFO discipline to 4.9 with the prioritization system. Table 4 shows how under the prioritization system the waiting time range among the regions was reduced as the priority score increased. Differences among patients with high priority scores were reduced substantially, while the results were uncertain in patients with medium or low priority scores.

The impact of introducing the prioritization system varied substantially among the regions studied, but reduced inequities among regions in patients with greater need. Figure 2 shows that the curves for Andalusia and Catalonia became closer as the priority score increases. Patients with a priority score of 40 had similar waiting times in both regions (4 months in Andalusia and 3 months in Catalonia), while differences in waiting time increased substantially in patients with priority scores of 20 (18 months in Andalusia and around 11 months in Catalonia). This pattern, however, was not observed when comparing the curves among Catalonia and Aragon, which maintained the differences among curves independently of priority score. To sum up, the prioritization system improves equity in patients with greater need, but not necessarily in all other patients.

In the present study, the variability found in surgery rates was not related to population characteristics or to the needs of the population on the waiting list. This lack of association indicates the need to improve the effectiveness of some management policies. Less than 6.5% of the population with need for surgery is included on a waiting list and there is wide variability in the priority scores assigned. Waiting lists do not represent unmet needs, but rather an auto-regulation mechanism of the health system. If the surgery supply is insufficient to cover unmet needs, it seems reasonable to introduce prioritization systems, which involve modifying the indication thresholds in accordance with the resources available in the system. The effectiveness of prioritization systems would increase substantially if prioritization was applied at surgery indication instead of assigning priorities only to patients entering the waiting list. If there is a substantial unmet need, clinicians could decide not to refer patients with low priorities for surgery, as they would have excessive waiting times. This fact would have an impact on the indication criterion. Giving a guarantee time to each patient related to his/her level of need would further increase equity, since levels of need in patients on the waiting list differ widely. Thus, the introduction of a prioritization system should entail an analysis of the unmet needs in each region, or at least involve a reduction in the variations in the surgery rates among regions.

The variables that appeared to have the greatest influence on the benefit obtained from the prioritization system and its impact in the waiting time were the variability in the priority scores at entry to the waiting list, the surgery rate and the waiting list volume. It is expected that the greater the waiting list and the lower the surgery rate within each region, the greater the benefit of introducing the prioritization system, as this would increase the waiting of patients and thus the benefit from introducing the prioritization system. Moreover, the higher the variability within each region in the priority scores assigned to patients, the higher the impact that can be expected from the prioritization system. If all patients had the same priority score, prioritization would have no impact.

Using data from the North London Eye Study might introduce some bias to the prevalence estimation. However a systematic review of cataract prevalence studies carried out by this research team[25] showed little differences in the prevalence by age among studies performed in several countries with populations similar to the Spanish population. This result minimizes the possible bias caused by assuming that the same cataract prevalence applies to North London and Spain. We assume that little differences in cataract prevalence would be found among Spanish regions because differences were small among international studies. The effect of the prioritization system might be overestimated because pure FIFO systems are rare and clinicians might use some implicit prioritization. However, a pilot study carried out by Espallargues et al.[14] found a slight prioritization in the Spanish cataract surgery waiting list. We defined several mathematical functions to approximate the relationships among certain parameters within the system. Thus, the quality of the information introduced in the model strongly depended on the quality of the information obtained from the different regions[16]. However, all the estimations made were validated by a panel of experts and consensus was reached by all regions' representatives. Some characteristics were estimated through data from other regions when access to the source of information was limited or information was unavailable.

## Conclusion

Discrete-event simulation is an appropriate and robust tool to study the impact and benefits of different health policy interventions in a context in which resources are scarce and there is wide variability in their management[16]. Introducing the prioritization system allows the impact of variations among regions to be reduced by improving the system's equity and effectiveness. On the one hand, effectiveness improves because patients with greater need have a shorter waiting time resulting in an overall saving of waiting time weighted by need. On the other hand, equity improves because the higher the need, the greater the reduction in differences in waiting time. However, the lower the priority, the greater increase in the differences among patients. The results of this study suggest that introducing the prioritization system would allocate the available resources within each region more efficiently.

## Competing interests

The author(s) declare that they have no competing interests.

## Authors' contributions

All the authors have contributed to the achievement of this study. RR participated in the design of the study, the statistical analysis and drafted the manuscript. MC participated in the design of the study, the statistical analysis and helped to draft and review the manuscript. JM, EB, AJ and SG helped in the design of the study and the review of the manuscript, participated in the validation of the model and provided essential information for the development of the analysis. XC conceived the study, and participated in its design and coordination and helped to draft and review the manuscript. All authors read and approved the final manuscript.

## Pre-publication history

The pre-publication history for this paper can be accessed here:


